# Idiopathic scrotal calcinosis: Case report and literature review

**DOI:** 10.1016/j.ijscr.2024.110770

**Published:** 2024-12-24

**Authors:** Xiaofeng Yang, Tao Tian, Qianqian Wang

**Affiliations:** a Department of Urology, Zaozhuang Municipal Hospital, 41th, Zaozhuang, China; b Department of Outpatient, Zaozhuang Municipal Hospital, 41th, Zaozhuang, China

**Keywords:** Idiopathic scrotal calcinosis, Scrotal mass, Surgery,Case report

## Abstract

**Introduction:**

This case report discusses the clinical manifestations, diagnosis and treatment of idiopathic scrotal calcinosis, and reviews the literature of similar cases, providing important reference for the diagnosis and treatment of this rare disease.

**Case presentation:**

Idiopathic scrotal calcinosis is a rare condition characterized by calcium deposition in the skin of the scrotum. We present a case of a 67-year-old male patient with idiopathic scrotal calcinosis, a rare condition characterized by calcium deposition in the skin of the scrotum. The patient initially noticed multiple nodular masses on his scrotum 30 years ago, which gradually increased in size and caused discomfort. Physical examination and enhanced CT scans revealed multiple, well-demarcated high-density nodules on the skin surface of the scrotum. The patient underwent surgical resection of the lesions, and histopathological findings confirmed the diagnosis of idiopathic skin calcium deposition in the scrotum.

**Discussion:**

Idiopathic calcinosis scrotum (ISC) is a rare benign surgical skin disorder that primarily affects the skin of the scrotum.It is characterized by the spontaneous deposition of calcium in the dermis, resulting in the formation of multiple asymptomatic calcified nodules. ISC is usually diagnosed based on clinical presentation and confirmed by histological examination. ISC can be treated well with surgery.

**Conclusion:**

Idiopathic Scrotal Calcinosis is a rare but distinct dermatosurgical disorder that requires surgical excision for definitive diagnosis and treatment. In managing ISC, surgical excision remains the primary treatment option, offering good outcomes and patient satisfaction. It is important for clinicians to recognize ISC to avoid misdiagnosis and unnecessary medical interventions.

## Highlights

• Idiopathic Scrotal Calcinosis is a benign disease that mainly affects young and middle-aged men.
• Surgery is the recommended treatment for relieving symptoms and improving beauty.
• Close follow-up is necessary to monitor for recurrence and manage any potential complications.
• Case reports help to understand and manage this rare skin disease.

## 1 Introduction

Idiopathic scrotal calcinosis is a rare and benign condition characterized by calcium deposition in the skin of the scrotum，which has only been reported in the hundreds worldwide [1,2]. The exact etiology of this condition remains unknown, and it typically presents as multiple, asymptomatic, hard nodules on the scrotal skin [3]. However, in some cases, the nodules may cause discomfort or become inflamed. Surgical resection is often the definitive treatment for idiopathic scrotal calcinosis. In order to better accumulate cases in this regard, the patient agreed to publish this case report. Case reports follow SCARE criteria [4].

## 2 Case presentation

**Fig. 1:**
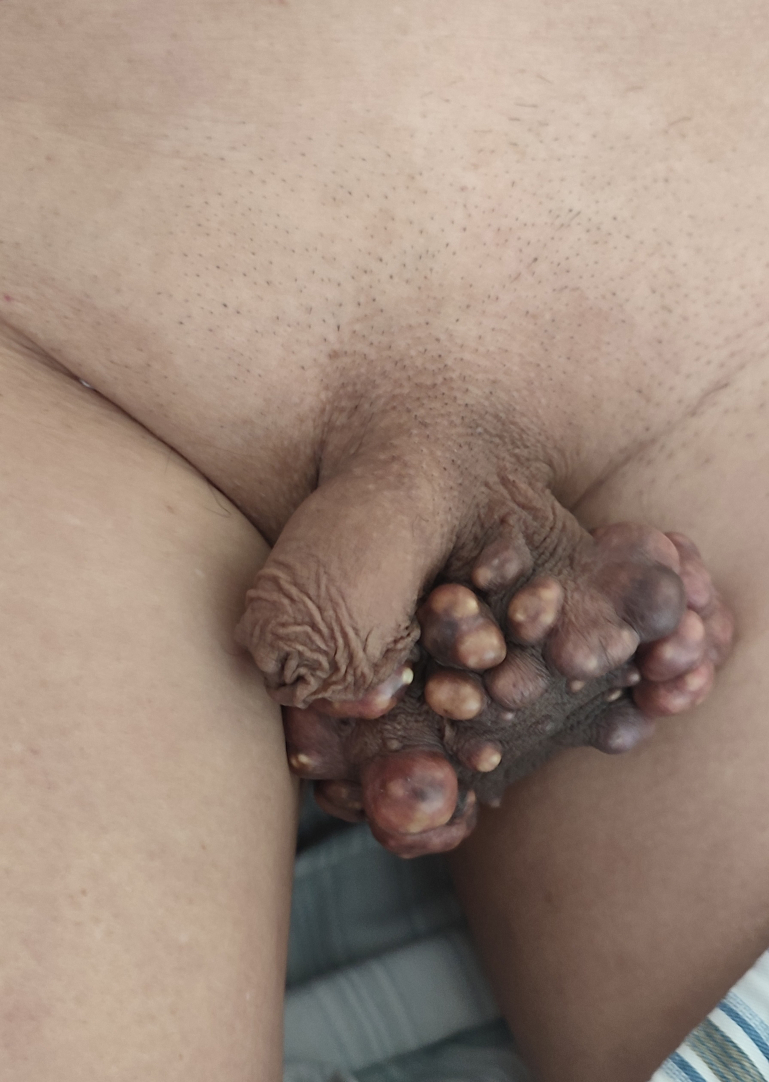
Clinical manifestations of skin lesions: Multiple yellow and white nodules of different sizes in the scrotum. (For interpretation of the references to colour in this figure legend, the reader is referred to the web version of this article.)

**Fig. 2:**
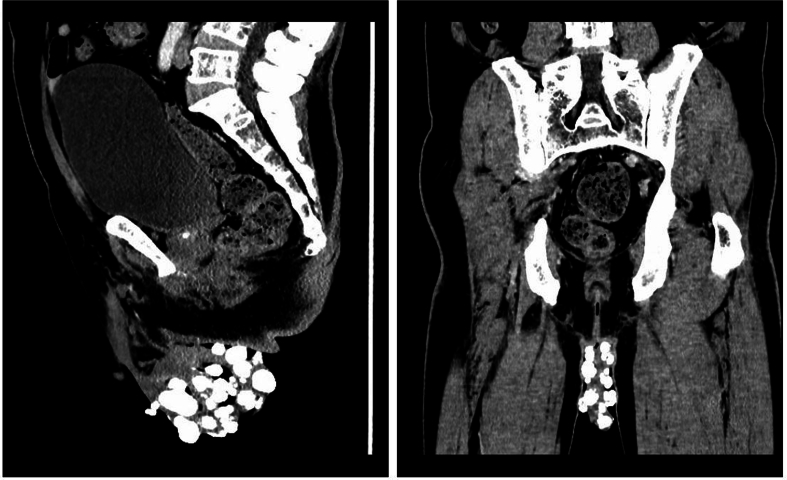
The coronal, sagittal and transversal CT views showed multiple exposed high-density nodules on the skin surface of the scrotum, with clear tubercle boundaries and the largest single tubercle size of 1.5 × 0.9 × 0.7 cm.

**Fig. 3:**
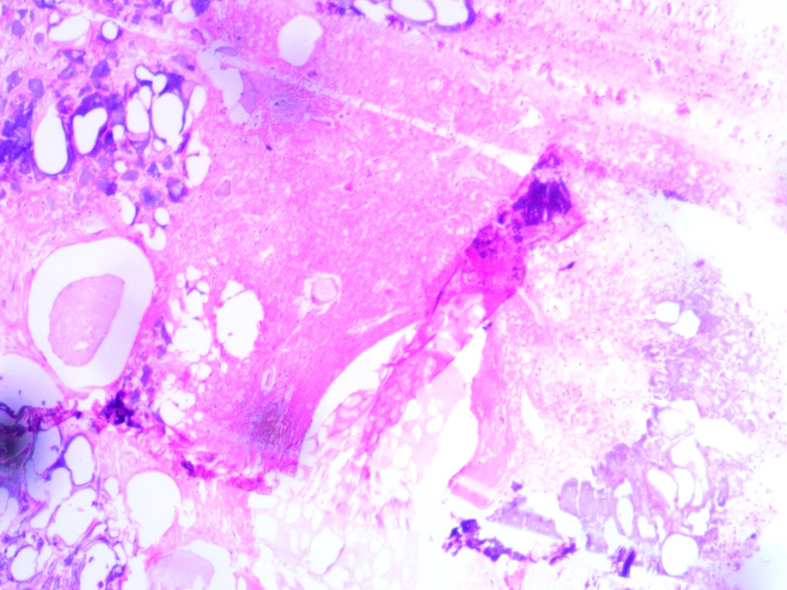
Histological sections stained with hematoxylin and eosin showed cystic lesions with calcium deposition.

A 67-year-old male patient presented to our hospital with a history of multiple nodular masses on his scrotum for 30 years. The nodules were initially small, about the size of “big rice grains,” and accompanied by itching. The patient noted that the nodules gradually increased in size and caused scrotal distension discomfort in the past 2 weeks. Physical examination revealed multiple yellow and white nodules distributed on the skin surface of the scrotum, varying in size and without obvious rupture or tenderness (Fig. 1). Enhanced CT scans showed diffuse thickening of the skin on both sides of the scrotum and multiple, well-demarcated high-density nodules, with the largest node measuring 1.5 × 0.9 × 0.7 cm (Fig. 2). The patient underwent scrotal lesion resection under general anesthesia. An incision was made about 0.5 cm from the edge of the mass, and part of the scrotum and the mass were completely removed. Histopathological findings revealed cystic lesions with calcium deposition, confirming the diagnosis of idiopathic skin calcium deposition in the scrotum (Fig. 3). The patient recovered well after surgery and had no recurrence at the one-month outpatient follow-up.

## 3 Discussion

Skin calcification denotes the accumulation of insoluble calcium salts within the dermis and subcutaneous tissues. Based on the underlying cause of calcium deposition, skin calcification can be categorized into five distinct types: dystrophic, metastatic, iatrogenic, calcific uremic arteriolopathy (also known as calcification defense), and idiopathic. The precise pathophysiological mechanisms underlying this phenomenon remain incompletely understood but may involve disruptions to collagen and the elastic fibers of the extracellular matrix, tissue microenvironment acidification stemming from injury and necrosis, alterations in calcium inhibitors, and abnormal elevations in mitochondrial calcium and phosphorus levels [5]. Dystrophic skin calcification is the most prevalent form, frequently observed in conditions such as systemic sclerosis and dermatomyositis. Its pathogenesis is linked to reduced blood supply or hypoxia during tissue injury, which stimulates an increase in intracellular calcium flux, leading to the deposition of insoluble calcium salts in damaged tissue and subsequent tissue mineralization. Notably, serum calcium and phosphorus levels remain within normal ranges in this type of calcification [6]. Metastatic skin calcification typically arises from chronic renal failure, which disrupts calcium and phosphorus metabolism, resulting in the deposition of calcium salts around joints [7]. Iatrogenic skin calcification commonly follows intravenous administration of calcium gluconate, calcium chloride, or phosphate-containing solutions that inadvertently extravasate. This is manifested by an increase in skin temperature at the injection site, accompanied by tender swelling, with the gradual formation of hard nodules within the dermis or subcutaneous tissue over time. Calcific uremic arteriolopathy (calcification defense) is characterized by calcification of dermal and subcutaneous arterioles, ultimately leading to skin tissue necrosis. This condition is predominantly seen in end-stage renal disease patients undergoing dialysis and can present as reticularis purpura, painful ulcers, and other symptoms [8].

Idiopathic Scrotal Calcinosis predominantly encompasses subepidermal calcifying nodules, tumor-like calcifications, and, of course, Idiopathic Scrotal Calcinosis itself [9]. The clinical presentation of subepidermal calcified nodules involves isolated, white, hard nodules typically located on the ears, neck, face, or upper limbs. Tumor-like calcinosis, on the other hand, commonly manifests as calcium deposits surrounding the shoulder, elbow, knee, and hip joints [10]. This specific condition, Idiopathic Scrotal Calcinosis, was initially described by Lewinski et al. in 1883 and later named by Shapiro et al. in 1970 [11]. It primarily affects men aged between 20 and 40 years. Idiopathic scrotal calcinosis is marked by a slow-growing, painless, yellowish nodule within the dermis of the scrotum, which may gradually enlarge and expand, occasionally accompanied by itching or chalky discharge. Patients typically seek medical attention for cosmetic reasons. The exact pathogenesis of this disease remains controversial, with ongoing debate about whether it is idiopathic or a form of dystrophic calcification stemming from rupture and degeneration of pre-existing structures (such as epidermal cysts, internal/exocrine epithelial cysts, and scrotal fascia muscle) following inflammation [12,13].

Idiopathic Scrotal Calcinosis (ISC) is an uncommon, benign dermatosurgical condition predominantly impacting the scrotal skin. It is distinguished by the spontaneous accumulation of calcium in the dermis, resulting in the development of multiple, asymptomatic calcified nodules [14]. Although ISC is categorized as a metabolic disorder, patients with this condition generally exhibit normal serum calcium levels [15]. The onset of ISC typically manifests during childhood or early adulthood, and its diagnosis is primarily based on clinical presentation, often corroborated by histological examination. Despite ongoing research, the precise pathogenesis of ISC remains partially understood, and while certain studies have hinted at a potential link with epidermal inclusion cysts, this connection has yet to be conclusively proven [16].

Idiopathic Scrotal Calcinosis typically manifests as multiple, asymptomatic, hard nodules on the scrotal skin, with a tendency to gradually enlarge over time. Occasionally, these nodules may cause discomfort or become inflamed, prompting patients to seek medical intervention. Diagnosis of idiopathic scrotal calcinosis is primarily based on clinical presentation, supported by imaging studies. During a physical examination, multiple, firm, non-tender nodules can be discerned on the scrotal skin. Imaging studies, notably CT scans, can clearly demonstrate calcium deposition within the nodules, thereby confirming the diagnosis. The management of idiopathic scrotal calcinosis primarily involves surgical resection of the affected lesions [17]. In our case, the patient underwent scrotal lesion resection under general anesthesia. Subsequent histopathological analysis confirmed the diagnosis of idiopathic skin calcium deposition in the scrotum. Following surgery, the patient recovered well and remained free of recurrence at the one-month follow-up.

Clinically, this disease should be distinguished from multiple lipocystoma of scrotum, calcified epithelial tumor, epidermoid cyst and scabies nodules. Idiopathic Scrotal Calcinosis refers to multiple yellowish and white nodules on the skin of the scrotum with no obvious cause or family history, and the lesions can break and discharge cheesy and chalky gravel particles, which has nothing to do with hormone and metabolic abnormalities.In this case, electrolytes such as calcium and phosphorus and parathyroid hormone were normal, and no cyst wall, mucosal epithelial tissue or lipid-like components were found in histopathology, which was different from multiple lipocystoma, sebaceous gland cyst, epidermal cyst or calcified epithelioma or adenoma. The diagnosis of Idiopathic Scrotal Calcinosis was considered in combination with the location of disease and histopathology. Previous literature reported that conservative treatment with diltiazem, tetracycline, aluminum hydroxide, colchicine, bisphosphonate, warfarin, intravenous immunoglobulin, etc. was effective for some patients [18]. There was no recurrence after laser or cryotherapy, but long-term observation was lacking and the number of cases was small [19]. Surgical resection is the gold standard of treatment, but postoperative recurrence is still reported, and the possible cause of recurrence is the presence of residual small calcified seeds in the skin of the scrotum at the time of the initial operation, which will increase with time [20]. Therefore, for patients with more skin lesions, complete local skin resection combined with scrotoplasty may be a better surgical choice, and partial resection may still recur.

## 4 Conclusion

Idiopathic scrotal calcinosis is an uncommon condition distinguished by the accumulation of calcium in the scrotal skin. It usually manifests as multiple, asymptomatic nodules, which may progressively enlarge and induce discomfort. Surgical resection stands as the definitive treatment for this condition, yielding favorable outcomes and low recurrence rates. Clinicians ought to enhance their diagnostic accuracy and differentiate it from multiple yellow and white sarcoidosis of the scrotum. Early and thorough surgical intervention is crucial to prevent any delays in treatment. Additional research is imperative to delve into the underlying causes and identify the optimal management strategies for idiopathic scrotal calcinosis.

## Ethical approval

Following approval of our Institutional Review Board and ethical committee, all procedures performed in studies involving human participants were in accordance with the ethical standards of the institutional and/or national research committee, and with the 1964 Helsinki Declaration and its later amendments or comparable ethical standards. The publication of the case report was approved by the Ethics Committee of the Zaozhuang Municipal Hospital，Zaozhuang, China on November 1, 2024.

## Guarantor

Qianqian Wang

## Research registration number

N/A

## Consent for publication

Written informed consent was obtained from the patient for publication of this case report and accompanying images. A copy of the written consent is available for review by the Editor-in-Chief of this journal on request.

## Declaration of Generative AI and AI-assisted technologies in the writing process

No.

## Funding

None.

## Declaration of competing interest

None.

## Data Availability

The manuscript contained all data related to this case report.

## References

[1] Shah V., Shet T. (2007 Apr). Scrotal calcinosis results from calcification of cysts derived from hair follicles: a series of 20 cases evaluating the spectrum of changes resulting in scrotal calcinosis. Am. J. Dermatopathol..

[2] Pontes G.H., Pinto C.T., Filho F.C. (2019). Idiopathic scrotal calcinosis: an unusual skin disorder[J].Revista Brasileira de Cirurgia Plástica (RBCP). Brazilian Journal of Plastic Sugery.

[3] Rimtebaye K., Ali Mahamat M., Kimassoum Rimtebaye F., Nemian M., Mouamba F.G., Djekoundade A., Andjefa V., Vadandi V., Mingue K., Noar T. (2022 Mar). Aspects cliniques anatomopatho- logiques et prise en charge de la calcinose scrotale [Clinical aspects and management of scrotal calcinosis]. Prog. Urol..

[4] Sohrabi C., Mathew G., Maria N., Kerwan A., Franchi T., Agha R.A., Collaborators. (2023 May 1). The SCARE 2023 guideline: updating consensus Surgical CAse REport (SCARE) guidelines. Int. J. Surg..

[5] Le C., Bedocs P.M. (2024 Jan). StatPearls [Internet].

[6] Del Barrio-Díaz P., Mellado-Francisco G., Vera-Kellet C. (2022 Jan 31). Generalized dystrophic calcinosis cutis in a patient with dermatomyositis. J. Gen. Intern. Med..

[7] Carr D.R., Pootrakul L., Chen H.Z., Chung C.G. (2019 Jan 1). Metastatic calcinosis cutis associated with a selective FGFR inhibitor. JAMA Dermatol..

[8] Gallo Marin B., Aghagoli G., Hu S.L., Massoud C.M., Robinson-Bostom L. (2023 Feb). Calciphylaxis and kidney disease: a review. Am. J. Kidney Dis..

[9] Jayarajah U., de Silva L., de Silva C., Seneviratne S. (2019 Dec 16). Idiopathic scrotal calcinosis: a case report of a rare entity. Case Rep. Urol..

[10] Zuo Q.Y., Cao X., Liu B.Y., Yan D., Xin Z., Niu X.H., Li C., Deng W., Dong Z.Y., Yang J.K. (2020 Feb). Clinical and genetic analysis of idiopathic normophosphatemic tumoral calcinosis in 19 patients. J. Endocrinol. Invest..

[11] Ding X., Ding S., Liu P. (2024). Idiopathic scrotal calcinosis[J]. Asian J. Surg..

[12] Qureshi P., Yaseen M.T., Bashir H. (2019). Pearls in the wrong pockets:idiopathic scrotal calcinosis[J]. Clin. Nucl. Med..

[13] Feng L., Shulin G., Jinhua W. (2022). Idiopathic calcinosis of scrotum:a case report and review of the literature[J]. Heliyon.

[14] Yuyucu Karabulut Y., Kankaya D., Şenel E., Dölek Y., Uslu A., Sertçelik A. (2015 Oct). Idiopathic scrotal calcinosis: the incorrect terminology of scrotal calcinosis. G. Ital. Dermatol. Venereol..

[15] Dubey S., Sharma R., Maheshwari V. (2010 Feb 15). Scrotal calcinosis: idiopathic or dystrophic?. Dermatol. Online J..

[16] Saad A.G., Zaatari G.S. (2001 Feb). Scrotal calcinosis: is it idiopathic?. Urology.

[17] Dubey S., Sharma R., Maheshwari V. (2010 Feb 15). Scrotal calcinosis: idiopathic or dystrophic?. Dermatol. Online J..

[18] Ali H., Buechler C.R., Lohr K.M. (2022 Aug 29). Calcinosis cutis: need for early and aggressive treatment. Rheumatol. Adv. Pract..

[19] Najar Seyf A., Alenmyr L. (2021). Scrotal calcinosis treated with carbon dioxide laser:two cases and a short literature review[J]. Acta Derm. Venereol..

[20] Sugihara T., Tsuru N., Kume H. (2014). Relapse of scrotal calcinosis 7 years after primary excision[J]. Urol. Int..

